# Supplementary material for: Active site specificity profiling datasets of matrix metalloproteinases (MMPs) 1, 2, 3, 7, 8, 9, 12, 13 and 14
Source: Data Brief. 2016 Feb 22;7:299–310. doi: 10.1016/j.dib.2016.02.036 (PMC4777984; doi:10.1016/j.dib.2016.02.036)
Supplement: Supplementary file 10 — Supplementary material [file mmc10.zip › WebPICS_hMMP3_G_1%/P1.html]

 

PICS results


|  |  |
| --- | --- |
| **P1\_A**  15 in 151 sites   9.9 %    effects > 10 perc. pnts.  (vice-versa in brackets)  P2\_Q: 10.7 (11.5)   P1prime\_Q: 13.4 (20.1)   P3prime\_G: 12.7 (17.4) |  |
| **P1\_C**  3 in 151 sites   2.0 %    effects > 10 perc. pnts.  (vice-versa in brackets)  P1prime\_V: 86.1 (12.3)   P3prime\_M: 30.0 (18.0) |  |
| **P1\_D**  15 in 151 sites   9.9 %    effects > 10 perc. pnts.  (vice-versa in brackets)  P1prime\_C: 13.4 (20.1)   P2prime\_I: 28.1 (23.4)   P3prime\_N: 12.7 (17.4) |  |
| **P1\_F**  6 in 151 sites   4.0 %    effects > 10 perc. pnts.  (vice-versa in brackets)  P2prime\_V: 53.5 (16.0)   P3prime\_N: 26.0 (14.2) |  |
| **P1\_H**  5 in 151 sites   3.3 %    effects > 10 perc. pnts.  (vice-versa in brackets)  P2\_S: 31.4 (12.1)   P1prime\_Y: 16.7 (16.7)   P2prime\_Q: 33.4 (16.7) |  |
| **P1\_K**  9 in 151 sites   6.0 %    effects > 10 perc. pnts.  (vice-versa in brackets)  P2\_G: 24.7 (17.1)   P2prime\_L: 43.0 (20.3)   P3prime\_G: 48.3 (39.5) |  |
| **P1\_N**  24 in 151 sites   15.9 %    effects > 10 perc. pnts.  (vice-versa in brackets)  P3\_I: 24.7 (45.6)   P3\_P: -24.3 (-13.6)   P2\_L: 13.7 (19.4)   P1prime\_I: -13.0 (-12.1)   P3prime\_A: 24.5 (22.6)   P3prime\_K: -12.4 (-11.9) |  |
| **P1\_Q**  13 in 151 sites   8.6 %    effects > 10 perc. pnts.  (vice-versa in brackets)  P2\_F: 11.4 (24.7)   P1prime\_V: 16.9 (10.4)   P2prime\_I: 26.6 (19.2) |  |
| **P1\_S**  14 in 151 sites   9.3 %    effects > 10 perc. pnts.  (vice-versa in brackets)  P2prime\_L: 23.1 (17.0)   P2prime\_R: 12.8 (13.8) |  |
| **P1\_Y**  7 in 151 sites   4.6 %    effects > 10 perc. pnts.  (vice-versa in brackets)  P2\_H: 10.3 (12.1)   P2\_Q: 33.6 (16.8)   P2prime\_C: 26.6 (62.1) |  |
